# Pyramiding Bacterial Blight Resistance Genes in Tainung82 for Broad-Spectrum Resistance Using Marker-Assisted Selection

**DOI:** 10.3390/ijms21041281

**Published:** 2020-02-14

**Authors:** Yu-Chia Hsu, Chih-Hao Chiu, Ruishen Yap, Yu-Chien Tseng, Yong-Pei Wu

**Affiliations:** 1 Department of Agronomy, National Chiayi University, Chiayi 60004, Taiwan; hsuychia@mail.ncyu.edu.tw (Y.-C.H.); ruishen0506@gmail.com (R.Y.); yct@mail.ncyu.edu.tw (Y.-C.T.); 2 Department of Agronomy, Chiayi Agricultural Experiment Station, Taiwan Agricultural Research Institute, Chiayi 60014, Taiwan; robinchiu3310@gmail.com

**Keywords:** rice, pyramiding, bacterial blight, marker-assisted selection, foreground selection, background selection

## Abstract

Tainung82 (TNG82) is one of the most popular *japonica* varieties in Taiwan due to its relatively high yield and grain quality, however, TNG82 is susceptible to bacterial blight (BB) disease. The most economical and eco-friendly way to control BB disease in *japonica* is through the utilization of varieties that are resistant to the disease. In order to improve TNG82’s resistance to BB disease, five bacterial blight resistance genes (*Xa4*, *xa5*, *Xa7*, *xa13* and *Xa21)* were derived from a donor parent, IRBB66 and transferred into TNG82 via marker-assisted backcrossing breeding. Five BB-resistant gene-linked markers were integrated into the backcross breeding program in order to identify individuals possessing the five identified BB-resistant genes (*Xa4, xa5*, *Xa7*, *xa13* and *Xa21*). The polymorphic markers between the donor and recurrent parent were used for background selection. Plants having maximum contribution from the recurrent parent genome were selected in each generation and crossed with the recipient parent. Selected BC_3_F_1_ plants were selfed in order to generate homozygous BC_3_F_2_ plants. Nine pyramided plants, possessing all five BB-resistant genes, were obtained. These individuals displayed a high level of resistance against the BB strain, XF89-b. Different BB gene pyramiding lines were also inoculated against the BB pathogen, resulting in more than three gene pyramided lines that exhibited high levels of resistance. The five identified BB gene pyramided lines exhibited yield levels and other desirable agronomic traits, including grain quality and palatability, consistent with TNG82. Bacterial blight-resistant lines possessing the five identified BB genes exhibited not only higher levels of resistance to the disease, but also greater yield levels and grain quality. Pyramiding multiple genes with potential characteristics into a single genotype through marker-assisted selection can improve the efficiency of generating new crop varieties exhibiting disease resistance, as well as other desirable traits.

## 1. Introduction

As a carbohydrate-rich staple of more than half the world’s diet, rice (*Oryza sativa* L.) is one of the most important food crops on the planet. The Food and Agriculture Organization of the United Nations (FAO) estimates that by 2050, overall global agricultural production may need to be increased by up to 70% to meet the dietary requirements of the world’s projected population of nine billion [1]. In order to satisfy the demand corresponding to the FAO’s projected population in 2050, global rice production would have to increase by nearly 42% over present-day levels [2]. Bacterial blight (BB) caused by *Xanthomonas oryzae* pv. *oryzae* (*Xoo*) is a disease that poses one of the greatest threats to rice production worldwide. In Asia, BB has proven to be capable of reducing crop yields by as much as 50% [3] to 80% [4]. The disease, being systemic, affects the photosynthetic areas of plants, which results in a drastically lower yield. Although BB can be managed through the use of fungicides, enhancing the genetic resistance in rice is the most effective and ecological method of overcoming the threat posed by the disease.

To date, 42 BB resistance genes have been identified from diverse sources, of which *Xa4*, *xa5*, *Xa7*, *xa13* and *Xa21* are most frequently utilized in BB resistance breeding programs [5,6,7]. Although the BB resistance genes *xa5* and *xa13* are recessive in nature, abundant molecular marker resources allow for molecular marker-assisted breeding [8,9,10,11]. The *xa5* gene encodes a mutated gamma subunit of basal transcription factor IIA 5 (TFIIAγ5), and along with the dominant resistance gene *Xa7*, has shown strong resistance to a virulent BB strain, Z-173, in China [11,12]. Another broad-spectrum recessive gene, *xa13,* was correlated with a plasma membrane protein conferring recessive resistance to PXO99 [13]. *Xa21,* which encodes a leucine-rich repeat (LRR) receptor kinase-type gene, was identified from *O. longistaminata*; it is one of the most effective genes utilized in breeding programs designed to enhance the BB resistance of rice cultivars [8,14].

Conventional backcross breeding embedded with marker-assisted selection (MAS) has been successfully employed in developing crop varieties exhibiting agronomically important traits. The utility of MAS in pyramiding several resistance genes to develop a variety possessing broad-spectrum durable resistance has been successfully demonstrated against numerous pathotypes [6,15,16]; *Jalmagna*, a high-yield, deep-water rice variety, was improved for BB resistance by pyramiding three resistance genes, *xa5* + *xa13* + *Xa21* [6]; a Korean elite *japonica* variety, *Mangeumbyeo*, improved with the introgression of the *Xa4* + *xa5* + *Xa21* genes, which were shown to possess a wide range of resistance to BB [16]. Recently, *xa5*, *xa13* and *Xa21* genes were introgressed into the hybrid rice maintainer lines CO2B, BO23B and CO24B through MAS, which can form the basis to develop new, widely adaptable heterotic hybrids possessing resistance against the destructive diseases to which rice is vulnerable [17]. In addition, there have been several examples of MAS being utilized to successfully incorporate different genes which provide higher resistance to various biotic and abiotic stresses (for example, the pyramiding of QTLs of submergence tolerance (*Sub1A*), leaf/neck blast (*qBL1* and *qBL11*), brown planthopper (*Bph3*) and BB (*xa5* and *Xa21*) in high-yielding and aromatic rice variety ‘Pink3′ [18]).

According to the annual report of the Council of Agriculture, of the 271,000 hectares of rice paddy fields in Taiwan, approximately 7% are affected by BB per year. Most Taiwanese *japonica* rice cultivars lack BB resistance genes [10], resulting in significant yield loss in fields severely infected by the disease. Pyramiding multiple *R* genes by MAS provides a rapid and precise way to develop a variety with wide-spectrum and durable resistance [19]. A set of 17 near-isogenic lines (NILs) in IR24 background, having single or two to four pyramided *Xa* genes, were included in the panel to serve as controls of known disease reactions [20]. IRBB66, carrying *Xa4*, *xa5*, *Xa7*, *xa13* and *Xa21*, in an *indica* rice IR24 genetic background, conferred strong resistance to races of BB. In the present study, five BB resistance genes were introgressed from IRBB66 into an elite *japonica* variety, ‘Tainung82′ (TNG82), using marker-assisted backcrossing (MAB) and marker-assisted background analysis of selected backcross progenies using SSR markers. The aims of this study were to (i) develop five gene pyramiding lines using MAB, (ii) evaluate the effects of BB-resistant lines carrying different R genes after inoculation with BB strain, (iii) select individuals possessing agronomic traits and grain quality performance from the resulting BB-resistant lines. The development of BB-resistant lines with more than three genes pyramided has a promising future in molecular breeding of durable BB-resistant rice cultivars.

## 2. Results

### 2.1. Development of BC_3_F_4_ Pyramided Lines Using Marker-Assisted Breeding

**Figure 1:**
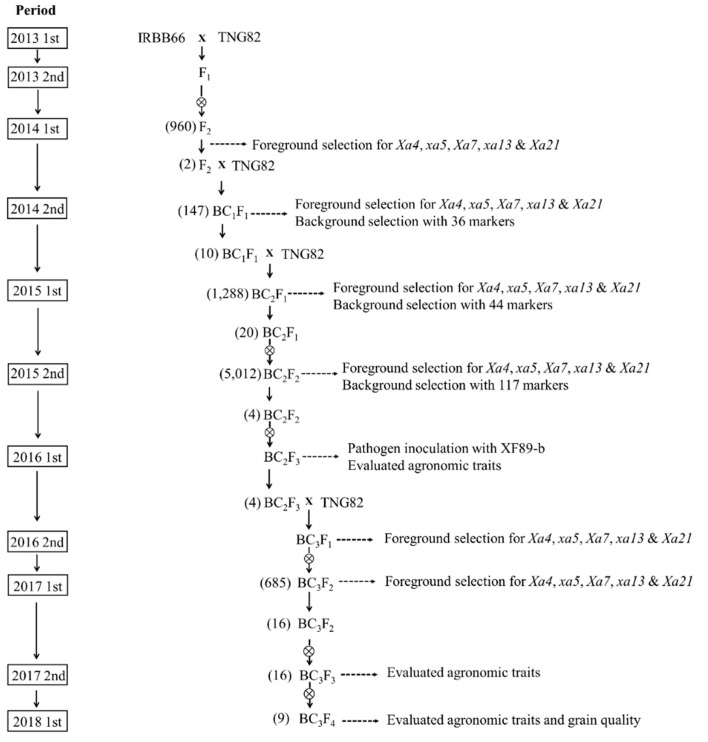
Schematic diagram for pyramiding bacterial blight resistance genes into Taiwanese *japonica* rice cultivar, TNG82, using marker-assisted selection and number of plants selected in every generation.

**Figure 2:**
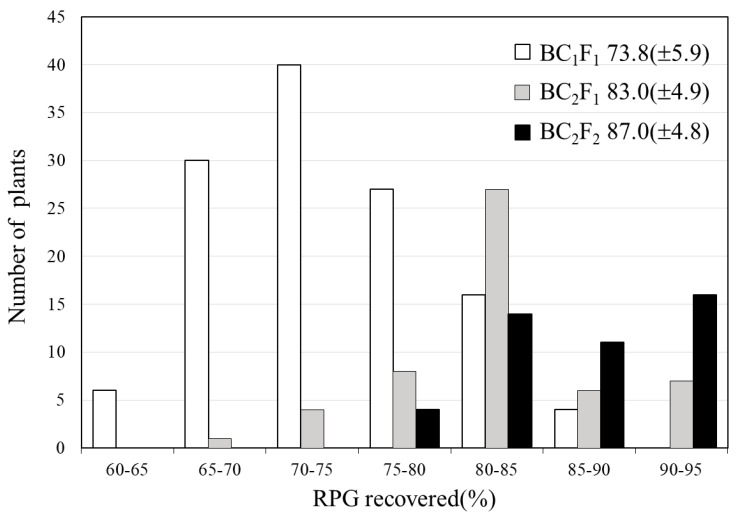
The frequency distribution of recurrent parent genome (RPG) recovered rate using marker-assisted backcrossing in BC_1_F_1_, BC_2_F_1_ and BC_2_F_2_ populations derived from the backcross of IRBB66/TNG82. The numbers inside the right side of frame indicate the mean values (SD) of RPG recovered.

Tainung82 is one of the most widely cultured elite *japonica* varieties in Taiwan, but it exhibits a high susceptibility to bacterial blight disease. In order to develop a BB-resistant *japonica* cultivar, TNG82 was used as the recurrent parent to backcross with IRBB66 for three generations, and then self-crossed to produce a BC_3_F_4_ population. The polymorphism was detected between donor parent IRBB66 and recurrent parent TNG82 with the markers Xa4F/4R, RM604F/604R, Xa7F/7-1R/7-2R, Xa13F/13R and Xa21F/21R for *Xa4*, *xa5*, *Xa7*, *xa13* and *Xa21*, respectively. In addition, the parents were screened with 216 rice microsatellite markers, of which 143 were polymorphic and 117 were used for background selection (Figure S1). The breeding scheme using molecular markers for the selection of the five BB-resistant genes is shown in Figure 1. During the breeding procedure, functional marker selection was practiced from the F_1_ generation until the BC_3_F_2_ generation. The plants possessing all five resistance genes were selected in each stage, of which only two progenies were advanced to the next generation. A total of two plants having all five BB resistance genes (*Xa4*, *xa5*, *Xa7*, *xa13* and *Xa21*) were screened from 960 F_2_ plants and confirmed by lined molecular markers [10]. The two F_2_ plants were backcrossed to TNG82. A total of 53 of 147 BC_1_F_1_ plants containing different BB resistance genes were selected by MAS. The percentages of recurrent parent genome (%RPG) of BC_1_F_1_ ranged from 60% to 85%, with an average of 73.8% (Figure 2). Ten BC_1_F_1_ plants containing both the five BB resistance genes, as well as a high %RPG (average of 81.7%), were used for further backcrossing with TNG82.

**Figure 3:**
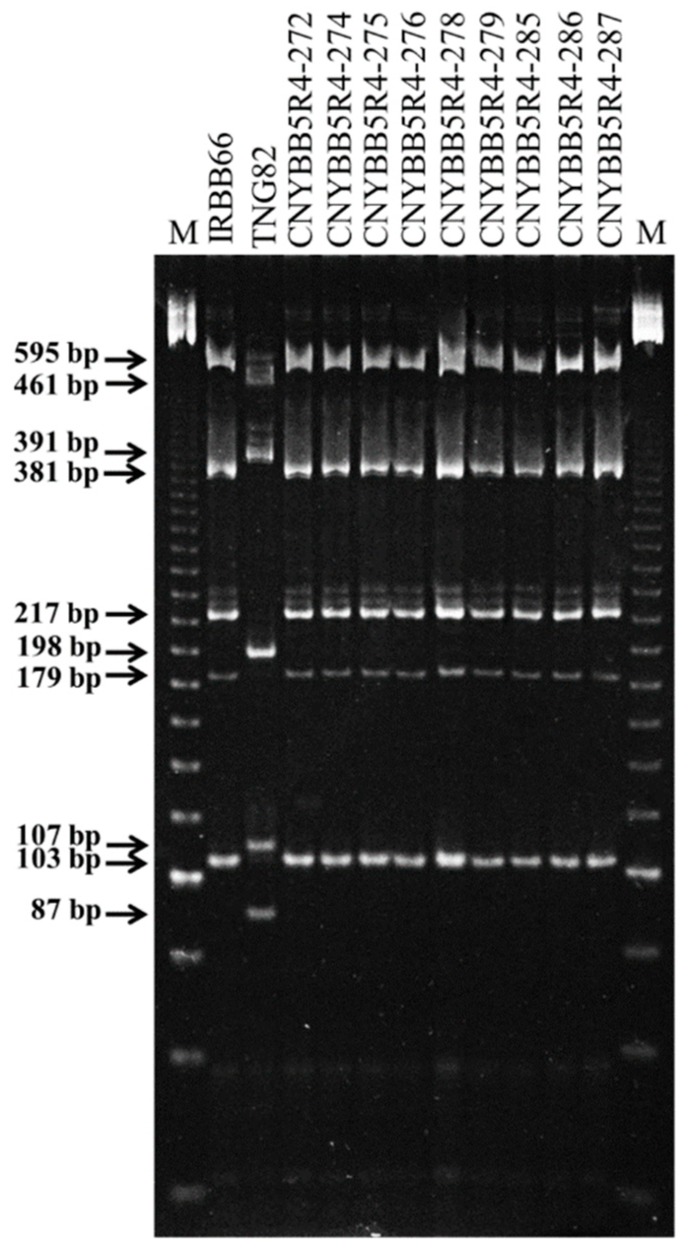
Multiplex PCR amplification of five bacterial blight resistance genes, *Xa4, xa5, Xa7, xa13* and *Xa21*. The five expected band sizes of 217, 106, 179, 381 and 595 bp, correlated with *Xa4, xa5, Xa7, xa13* and *Xa21*, respectively, were amplified in IRBB66 and nine five-gene-pyramided lines using multiplex PCR. P1:IRBB66, P2:TNG82. DNA products were separated by 6% polyacrylamide gel in 0.5 × TBE at 100 v for 60 min. M: DNA ladder marker.

A total of 50 of 1228 BC_2_F_1_ plants containing different BB resistance genes possessed the recurrent genome content of TNG82, ranging from 72% to 94%, with an average of 83% (Figure 2). The 20 selected BC_2_F_1_ plants, heterozygous for all five BB resistance genes and possessing a high %RPG (average of 87.3%), were selfed to obtain the BC_2_F_2_ population. The plants homologous for all five target genes were segregated with a Mendelian pattern (homozygous preference genotype = 1/4^n^). The four BC_2_F_2_ plants carrying five positive homozygous alleles of the donor genes, including *Xa4*, *xa5*, *Xa7*, *xa13* and *Xa21*, were screened from 5012 BC_2_F_2_ plants. Four BC_2_F_2_ plants showed recurrent genome content of TNG82 with %RPG of 92.05% (29), 84.3% (18), 83.1% (5) and 79.4% (43), with an average of 84.71% (Table S1). In the BC_2_F_3_ generation, 17 plants containing different BB resistance genes were used to confirm resistance reaction by inoculation with *Xoo* isolate XF89-b and evaluated for agronomic performance. Four BC_2_F_3_ plants with the five BB resistance genes were backcrossed to TNG82. In the BC_3_F_2_ generation, 16 of 685 plants containing the five BB resistance genes were identified and grown as BC_3_F_3_. These 16 five-gene-pyramided genotypes were selfed and evaluated for agronomic performance. The nine BC_3_F_4_ lines containing five BB resistance genes, *Xa4*, *xa5*, *Xa7*, *xa13* and *Xa21* (Figure 3) were selected and evaluated for agronomic performance in the field, as well as analyzed for grain quality.

### 2.2. Development of BC_3_F_4_ Pyramided Lines Using Marker-Assisted Breeding

**Figure 4:**
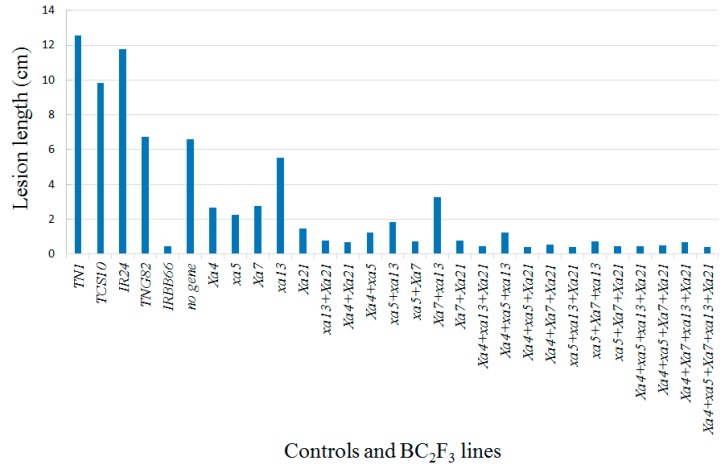
The leaf lesion length of TNG82/IRBB66 BC_2_F_3_ genotypes after 21 days inoculum of bacterial blight pathogen XF89-b in the field at the first crop season in 2016. Susceptible cultivar: TCS10, IR24, and TN1; Parental: TNG82 and IRBB66.

**Figure 5:**
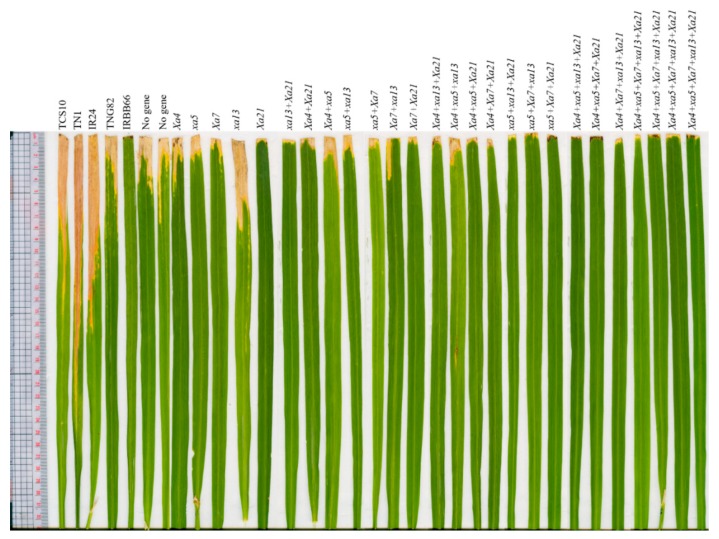
The leaf lesion photo of TNG82/IRBB66 BC_2_F_3_ genotypes after 21 days inoculum of bacterial blight pathogen XF89-b in the field at the first crop season in 2016. Susceptible cultivar check: TCS10, IR24, and TN1; Parents: TNG82 and IRBB66.

The BC_2_F_3_ pyramided rice genotypes were evaluated for their resistance to BB in the field conditions using the Taiwanese *Xanthomonase oryzae* strain isolate, XF89-b. The resistance donor IRBB66, containing five BB resistance genes, showed shorter lesion lengths (mean lesion length of 0.43 cm), while the susceptible checks, TN1, TCS10, IR24 and TNG82, exhibited a range of longer lesion lengths, between 6.75 and 12.56 cm (Table 1, Figure 4). The genotypes having either BB resistance genes alone or more than two genes pyramided were shown to be moderately resistant, resistant, and highly resistant to the BB disease (Figure 5). In addition, the five-gene-pyramided BC_2_F_3_ genotypes exhibited a range of shorter lesion lengths, between 0.37 and 0.46 cm (Table 1). The five-gene-pyramided lines displayed higher levels of disease resistance and a broader resistance spectrum compared to both the parental rice variety, TNG82 and the genotypes possessing a single gene.

### 2.3. Development of BC_3_F_4_ Pyramided Lines Using Marker-Assisted Breeding

**Figure 6:**
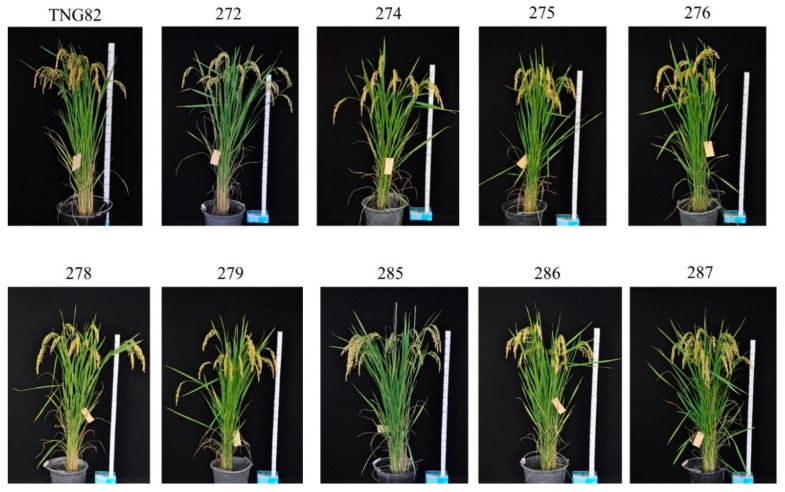
Phenotype of the five-gene-pyramided BC_3_F_4_ genotypes compared with recurrent parental variety TNG82.

Nine five-gene-pyramided lines at the BC_3_F_4_ generation, along with the recurrent and donor parents, were evaluated in the first crop season of 2018 at Taiwan Agricultural Research Institute (TARI), Taiwan. Significant variances were observed between the pyramided lines and parental rice genotypes for plant height, days to 50% flowering, panicle length, panicles/plant, panicle weight, number of grains/panicle, 1000-seed weight, and single plant yield (Table 2). The recurrent parent, TNG82, recorded mean grain yield of 36.8 g/plant, while the donor parent, IRBB66, was 30.1 g/plant. Six of the nine five-gene-pyramided lines, CNYBB5R4-275, -276, -278, -279, -285 and -287, produced significantly higher grain yields per plant than the recurrent parent, which ranged from 37.1 to 44.5 g/plant, and displayed a similar phenotype to the donor parent TNG82 (Figure 6).

A significant difference was noted between the parental rice varieties and pyramided genotypes in grain quality traits (Table 3). The palatability among pyramided BC_3_F_4_ genotypes varied between 69.8 (CNYBB5R4-275) and 74.5 (CNYBB5R4-276). The protein content among pyramided BC_3_F_4_ genotypes varied between 6.4 (CNYBB5R4-276 and CNYBB5R4-286) and 7.4 (CNYBB5R4-272). The brown rice ratio for the five-gene-pyramided genotypes varied from 72.8% to 79.3%. The four genotypes, CNYBB5R4-272, -275, -276 and -278, were found to have higher head rice ratios, however, the amount of total milled rice was not significantly different from the recurrent parent, TNG82. The evaluation of agronomic traits in BC_3_F_3_ and BC_3_F_4_ provided us with an important selection criteria, which can select candidate lines with stable agronomic performances and resistance to disease.

## 3. Discussion

Conventional backcross breeding is the primary method used to develop highly BB-resistant rice cultivars, but it cannot accurately transfer multiple genes into the cultivar by phenotypic screening and the process requires a significant amount of time [21,22]. Modified backcross pyramid breeding, combined with molecular marker-assisted selection, has already been demonstrated to increase the precision and efficiency of breeding [23,24,25]. Due to the relatively large amount of work involved with the MAS process, the conventional backcross breeding approach has been widely adopted in breeding programs designed to breed for BB resistance [10,26,27,28].

To date, many rice cultivars with broad-spectrum resistance against *Xoo* isolates have been developed; Singh et al. (2001) pyramided three *R* genes, *xa5*, *Xa13* and *Xa21*, in the *indica* rice cultivars PR106 and Jalmagna using MAS to enhance the bacterial blight resistance [6,15]; the four genes *Xa4*, *xa5*, *Xa13* and *Xa21* were introgressed into the recurrent parent lines Jyothi, IR50, Mahsuri, PRR78, KMR3 and Pusa 6B [26,29,30]; different BB-resistant genes, *Xa7*, *Xa21*, *Xa22* and *Xa23*, were also transferred to an elite hybrid rice restorer line, Huahui 1035, in order to improve BB resistance and enhance rice yield [31].

In Taiwan, many *japonica* rice cultivars lack BB resistance, resulting in significant yield loss in severely infected fields. One such variety is Tainung82, which was released in Taiwan for commercial cultivation in 2006. TNG82 is described as a popular *japonica* rice variety, with high-yield potential (6–7 t/ha), excellent grain quality, various culinary applications, and relatively low grain protein content (4.5%–5.5%). As an extremely valuable yet BB-susceptible variety, TNG82 was selected as the focus of this study to increase BB resistance through the introgression of five BB-resistant genes, *Xa4*, *xa5*, *Xa7*, *xa13* and *Xa21*.

The primary purpose of backcross breeding is to transfer one or multiple genes of interest, linked to desirable traits, from donor parents into a base variety for improvement, a process which typically requires six to eight backcrosses to recover the recurrent parent’s phenotype [32]. However, in the MAS scheme, three to four generations of backcrossing is generally enough to achieve more than 99% of the recurrent parent genome [33]. The theoretical %RPG of each generation, BC_1_, BC_2_, BC_3_ and BC_4_, were 75%, 87.5%, 93.8% and 96.9%, respectively. Furthermore, the %RPG can be improved by using MAS for background selection [16,34]. The 80% and 89% recovery rates following two and three backcrosses were obtained from three-BB-gene-pyramided BC_2_ and BC_3_ genotypes, via MAS [35]. Balachiranjeevi et al. (2015) transferred the BB gene, *Xa21* and rice blast-resistant gene, *Pi54,* to DRR17A and were able to recover 73.4%, 84.8% and 93.4% RPG in the BC_1_, BC_2_ and BC_3_ generations, respectively.

In this study, the recurrent parent genome recovery rates in BC_1_F_1_, BC_2_F_1_ and BC_2_F_2_ were 73.8%, 83% and 84.7% (Figure 2), respectively. Compared with the theoretical %RPG, a relatively low background recovery rate was obtained, however, the results were consistent with those found in previous studies [36,37]. Marker-assisted backcrossing can accelerate the breeding process and facilitate a speedy recovery for most of the recurrent genome within a few generations [38], however, the population size of each backcross generation, linkage drag, number of background markers used and genetic background between two parents are considered to be factors that reduce the efficiency of MAB and %RPG [32].

Bacterial blight is one of the most destructive diseases affecting rice productivity in Asia. In Taiwan, rice production is frequently affected by BB in the second crop season, resulting in substantial yield loss. In recent years, BB has become a more prevalent threat, due to climate change [39]. XF89-b, a strong and stable Taiwanese epidemic pathogen, has also been used for genetic analysis and the mapping of BB-related resistance genes [40]. In our bioassays, artificial screening of BC_2_F_3_ progenies revealed that all genotypes containing at least one BB-resistant gene displayed a degree of increased resistance (Table 1, Figure 4). The BC_2_F_3_ progenies that pyramided more than three BB-resistant genes exhibited a very high level of BB resistance against the XF89-b strain, compared to parental lines (Figure 5). The lesion lengths were measured between 0.37 and 1.25 cm (Table 1). The data indicated that multiple BB-resistant genes pyramided in rice can improve resistance to *Xoo*. The BB pyramiding lines are expected to enhance the adaptability and durability necessary to provide resistance against the dynamic nature of the pathogen. In addition, the results suggest that the gene combinations containing the *Xa21* gene were most resistant, as evidenced by shorter lesions lengths, followed by *Xa4* + *Xa21*, *Xa7* + *Xa21* and *xa13* + *Xa21*, while lines with *Xa4 + xa5*, *xa5* + *xa13* and *Xa7 + xa13* were less effective. These results are consistent with previous studies, which have shown the presence of *Xa21* to be correlated with high levels of persistent resistance against BB disease in rice [6,14,15,17,25]. *Xa21* is the cell surface receptor, kinase, which is able to provide resistance to *Xoo* infections; *Xa21* not only suppresses *Xoo* growth, but also triggers broad perturbation in rice transcriptomes and mediated signaling pathways, preventing *Xoo* infections [14].

The agronomic performance evaluation of BC_3_F_4_ derived in the genetic background of TNG82 revealed that all pyramided lines for most of the agro-morphological traits were, in general, similar to the recipient parent, TNG82. However, six candidate lines, CNYBB5R4-275, -276, -278, -279, -285 and -287, produced significantly higher grain yields per plant than the recurrent parent, which was further confirmed by the multilocation evaluation. In addition, three candidate lines, CNYBB5R4-276, -278 and -286, were not significantly different in palatability, protein, amylose, brown rice ratio, head rice ratio or total milled rice ratio, indicating that the BC_3_F_4_ pyramiding lines had grain quality consistent with TNG82. The data showed that there were no yield or grain quality reductions, but rather improvements, due to the pyramiding of the five BB-resistant genes.

## 4. Materials and Methods

### 4.1. Plant Materials

The donor parent, IRBB66, contained five resistance genes, *Xa4, xa5, Xa7, xa13* and *Xa21*, which were introgressed from wild species in the background of IR24. IRBB66 was provided as a courtesy by the Genetic Resources Center (GRC) of the International Rice Research Institute (IRRI). The recurrent parent was TNG82, an elite japonica cultivar with low protein content and good grain quality, but susceptible to bacterial blight disease. A cross was made between TNG82 and IRBB66, with F_1_ plants backcrossed thrice with TNG82 to obtain BC_3_F_1_ plants, which were selfed to obtain the BC_3_F_4_ progeny. Selection based on foreground, background and agronomic traits were practiced from BC_1_F_1_ to BC_2_F_2_ as a means of identifying lines similar to the recurrent parent.

### 4.2. Evaluation of Bacterial Blight Resistance

The parental varieties (IRBB66 and TNG82), susceptible varieties (Taichung Native 1 (TN1), Taichung sen 10 (TCS10)), BC_2_F_2_ and BC_2_F_3_ generation genotypes were pyramided with the five BB-resistant genes, with IR24 as control. Different combinations were evaluated for BB resistance under greenhouse and field conditions with the pathogen, *X. oryzae* pv. *oryzae*. Pathogen inoculation was performed at the maximum tillering stage in the field through the modified leaf clipping method, as previously described [41]. A strong Taiwanese epidemic pathogen isolate, XF89-b, was used in this study. The isolate was grown in 523 medium [42] with agitation at room temperature for two days. After adjusting the optical bacterial density to 10^9^ CFU/mL with distilled water, the cultures were used to screen the rice plants for BB resistance. Approximately six leaves from one plant were clipped from the top 2–3 cm simultaneously. All inoculation was completed within 1 h following the preparation of bacterial suspensions. Lesion length for BB was scored after inoculation when the lesion of the susceptible variety, TN1, reached approximately 3/4 of overall leaf length (approximately 21–28 days). The resistance reaction was classified as highly resistant (HR), resistant (R), moderately resistant (MR), moderately susceptible (MS), and susceptible (S) when the values of lesion length were recorded as 0–1 cm, 1.1–3 cm, 3.1–6 cm, 6.1–10 cm, and more than 10 cm, respectively [43,44].

### 4.3. Evaluation of Agronomic Traits

During the second and first crop season of 2017 and 2018, respectively, the 30-day-old seedlings of the BC_3_F_3_ and BC_3_F_4_ pyramided lines and both the parents were transplanted into three rows, with 27 plants per row, per entry, at 15 × 25 cm spacing, under a randomized complete-block design, with two replications at the Taiwan Agricultural Research Institute’s Chiayi Agricultural Experiment Station Farm. Ten plants from each entry were recorded as one data replication. Single plant yield was recorded for the 16 BC_3_F_3_ genotypes as a basis for selection. In BC_3_F_4_, variables for agronomic traits were recorded for nine pyramided lines, including: plant height (cm), days to 50% flowering, panicle length (cm), panicles/plant, panicle weight (g), number of grains/panicle and 1000-seed weight (g), while single plant yield (g) was recorded on a whole-plot basis. In addition, the grain quality, including palatability, protein, amylose, brown rice ratio, head rice ratio and total milled rice ratio, was investigated and analyzed. For palatability analyses, the rice grains were hulled and ground into a fine flour. Approximately 33 g of rice flour was used for the palatability evaluation, which was performed by using a palatability analyzer system (Toyo Taste Meter, Model MA-30), in accordance with the manufacturer’s operation manual (TRCM Co., Toyo Rice Polishing Machine Factory, Japan), as previously described [45]. Protein and amylose contents were measured with a near-infrared spectrometer (AN820, Kett Electric Laboratory Co. Ltd., Tokyo, Japan) (Near Infrared Spectrometer, Foss Japan Co. Ltd., Tokyo, Japan). Statistical analysis was performed with independent samples using least significant difference (LSD).

### 4.4. DNA Isolation and PCR Amplification

A rice genomic DNA extraction, with modification, was adopted for minipreparation [45]. Approximately 0.05 g of fresh leaf tissue from 6- to 8-week-old seedlings was homogenized with 300 μL extraction buffer (100 mM Tis-HCl, pH 9.0; 40 mM EDTA-2Na, pH 8.0; 1.67% SDS) at 30 1/s for 2 min by use of TissueLyser (Qiagen Retsch GmbH, Haan, Germany). A total of 150 μL benzyl chloride was added to the homogenized tissue and vortexed. After incubation in a 50 °C water bath for 15 min, 150 μL of 3 M sodium acetate (pH 5.2) was added. Supernatants were saved after centrifugation at 15,000 rpm for 15 min at 4 °C, and 300 μL of ice-cold isopropanol was added to precipitate DNA. After centrifugation at 15,000 rpm for 10 min, DNA pellets were saved and washed with 70% ethanol, air-dried and dissolved in 50 μL TE buffer.

A 10 μL PCR reaction containing 20 ng genomic DNA, 0.2 μM forward and reverse primers, 5 μL Multiplex PCR Master Mix (QIAGEN, Inc., Valencia, CA), and 1 μL Q-Solution (QIAGEN, Inc., Valencia, CA) was performed by use of a thermocycler (GeneAmp PCR System 9700, PerkinElmer Corp., Norwalk, CT, USA) at 95 °C for 15 min for 1 cycle; 94 °C for 30 s, 57 °C for 2 min, 72 °C for 2 min for 30 cycles; and 60 °C for 30 min for 1 cycle. Following PCR, 2 μL of amplified DNA products was separated by 6% polyacrylamide gel (PAGE) in 0.5 × TBE at 100 v (Dual Triple-Wide Mini-Vertical System, C. B. S. Scientific, CA, USA) for 60 min.

### 4.5. Marker Analysis

Five gene-specific primers, Xa4F/4R, RM604F/604R, Xa7F/7-1R/7-2R, Xa13F/13R, and Xa21F/21R, tightly linked to the resistance genes *Xa4, xa5, Xa7, xa13* and *Xa21*, respectively, were used to confirm the presence of the R genes in each generation. All markers in this study were published in the previous report [10]. In addition, a total of 36 and 44 markers of known chromosomal positions were used for genotyping in BC_1_F_1_ and BC_2_F_1_, respectively. In BC_2_F_2_, 117 markers, including 57 SSRs, 9 STS, and 51 InDel, distributed evenly on the 12 chromosomes with an average marker interval of 12.76 cM, were used in a genome-wide survey to identify the chromosome segment substitution locations. These polymorphic markers were used for background selection in order to select plants having maximum recovery of the recurrent parent genome. The genotypes from polymorphic bands are recorded as A (IRBB66), B (TNG82) and H (IRBB66/TNG82). The Graphical Geno Types (GGT) Version 2.0 [46] software program was used for the assessment of the recurrent parent genome (%RPG) in the selected recombinants, based on marker data.

## 5. Conclusions

The use of marker-assisted selection in backcross breeding is an effective and reliable approach for pyramiding BB-resistant genes in rice. In this study, the pyramiding lines that possess resistance against BB strains, high potential yields, and high grain quality were both developed and improved. The BB-pyramided breeding lines containing all five genes, *Xa4, xa5, Xa7, xa13* and *Xa21*, can serve as donors to introgress the resistance genes into other elite rice cultivars in order to accelerate the improvement of rice for disease resistance in Taiwan. These BB-pyramided lines are expected to have a high impact on domestic rice production stability, and also reduce the need for pesticides.

## Figures and Tables

**Table 1:** The results of TNG82/IRBB66 BC_2_F_3_ lines after incubating the *Xoo* strains XF89-b in the field at the first crop season in 2016.

No.	Lines	Genotypes	Lesion Length ^†^ (cm)	Resistance Scale ^¥^
1	TN1		12.56 ± 2.98 ^a^	S
2	TCS10		9.85 ± 2.07 ^b^	MS
3	IR24		11.75 ± 1.80 ^a^	S
4	TNG82		6.75 ± 2.54 ^c,d^	MS
5	IRBB66	*Xa4 + xa5 + Xa7 + xa13 + Xa21*	0.43 ± 0.70 ^i^	HR
6	CNYBB0R01	Without resistance gene	7.30 ± 1.50 ^d^	MS
7	CNYBB0R02	Without resistance gene	5.91 ± 2.04 ^c^	MR
8	CNYBB1R01	*Xa4*	2.67 ± 1.00 ^e,f^	R
9	CNYBB1R02	*xa5*	2.27 ± 0.73 ^e,f,g^	R
10	CNYBB1R03	*Xa7*	2.77 ± 1.04 ^e,f^	R
11	CNYBB1R04	*xa13*	5.55 ± 2.28 ^d^	MR
12	CNYBB1R05	*Xa21*	1.48 ± 1.48 ^f,g,h,i^	R
13	CNYBB2R03	*xa13 + Xa21*	0.77 ± 0.45 ^h,i^	HR
14	CNYBB2R04	*Xa4 + Xa21*	0.68 ± 0.24 ^h,i^	HR
15	CNYBB2R05	*Xa4 + xa5*	1.24 ± 0.98 ^g,h,i^	R
16	CNYBB2R06	*xa5 + xa13*	1.82 ± 0.54 ^f,g,h^	R
17	CNYBB2R01	*xa5 + Xa7*	0.75 ± 0.33 ^h,i^	HR
18	CNYBB2R07	*Xa7 + xa13*	3.25 ± 0.73 ^e^	MR
19	CNYBB2R02	*Xa7 + Xa21*	0.76 ± 0.19 ^h,i^	HR
20	CNYBB3R03	*Xa4 + xa13 + Xa21*	0.45 ± 0.17 ^h,i^	HR
21	CNYBB3R04	*Xa4 + xa5 + xa13*	1.25 ± 0.74 ^g,h,i^	R
22	CNYBB3R05	*Xa4 + xa5 + Xa21*	0.46 ± 0.17 ^h,i^	HR
23	CNYBB3R01	*Xa4 + Xa7 + Xa21*	0.56 ± 0.16 ^h,i^	HR
24	CNYBB3R06	*xa5 + xa13 + Xa21*	0.44 ± 0.08 ^h,i^	HR
25	CNYBB3R07	*xa5 + Xa7 + xa13*	0.71 ± 0.45 ^h,i^	HR
26	CNYBB3R02	*xa5 + Xa7 + Xa21*	0.44 ± 0.12 ^h,i^	HR
27	CNYBB4R03	*Xa4 + xa5 + xa13 + Xa21*	0.43 ± 0.12 ^i^	HR
28	CNYBB4R01	*Xa4 + xa5 + Xa7 + Xa21*	0.48 ± 0.17 ^h,i^	HR
29	CNYBB4R02	*Xa4 + Xa7 + xa13 + Xa21*	0.68 ± 0.50 ^h,i^	HR
30	CNYBB5R01	*Xa4 + xa5 + Xa7 + xa13 + Xa21*	0.37 ± 0.13 ^i^	HR
31	CNYBB5R02	*Xa4 + xa5 + Xa7 + xa13 + Xa21*	0.38 ± 0.12 ^i^	HR
32	CNYBB5R03	*Xa4 + xa5 + Xa7 + xa13 + Xa21*	0.46 ± 0.14 ^h,i^	HR
33	CNYBB5R04	*Xa4 + xa5 + Xa7 + xa13 + Xa21*	0.38 ± 0.12 ^i^	HR

^†^ Mean ± standard error. ^¥^ HR = highly resistant (lesion length < 1 cm); R = resistant (1 cm < lesion length < 3 cm); MR = moderately resistant (3 cm < lesion length < 6 cm); MS = moderately susceptible (6 cm < lesion length < 10 cm); S = susceptible (10 cm < lesion length). Means with none or the same letter of a row are not significantly different at 5% level by least significant difference (LSD) test.

**Table 2:** Agro-morphologic traits of parental and five-gene-pyramided BC_3_F_4_ genotypes.

Pyramided Lines	Plant Height (cm) (*n* = 20)	Days to 50% Flowering (*n* = 20)	Panicle Length (cm) (*n* = 20)	Panicles/Plant (*n* = 20)	Panicle Weight (g) (*n* = 20)	No. of Grains/Panicle (*n* = 20)	1000-Seed Weight (g) (*n* = 20)	Single Plant Yield (g) (*n* = 20)
TNG82	105.4 ± 1.1	90	20.3 ± 0.2	14 ± 0.7	3.3 ± 0.1	102 ± 2.5	30.8 ± 0.1	36.8 ± 0.1
IRBB66	90.2 ± 1.4	95	22.6 ± 0.1	17 ± 0.5	3.7 ± 0.1	108 ± 1.0	24.2 ± 0.1	30.1 ± 0.7
CNYBB5R4-272	120.4 ± 1.3	92	22.8 ± 1.8	16 ± 0.8	3.6 ± 0.4	122 ± 16.7	26.0 ± 0.1	36.3 ± 7.3
CNYBB5R4-274	115.9 ± 2.7	91	21.4 ± 1.6	15 ± 0.5	2.8 ± 0.1	95 ± 8.2	28.4 ± 0.4	32.6 ± 4.4
CNYBB5R4-275	116.3 ± 3.7	91	20.1 ± 0.6	16 ± 2.1	3.2 ± 0.3	113 ± 17.8	25.2 ± 0.4	41.2 ± 3.3
CNYBB5R4-276	116.3 ± 3.2	91	20.4 ± 0.7	16 ± 0.5	2.8 ± 0.2	100 ± 9.9	26.4 ± 0.4	43.7 ± 0.9
CNYBB5R4-278	115.8 ± 2.4	91	21.7 ± 0.2	12 ± 0.8	3.9 ± 0.3	135 ± 10.3	26.2 ± 0.1	44.5 ± 1.3
CNYBB5R4-279	112.7 ± 4.2	91	18.3 ± 0.1	15 ± 1.7	3.1 ± 0.1	120 ± 4.7	25.2 ± 0.2	39.2 ± 3.0
CNYBB5R4-285	121.8 ± 3.0	92	21.6 ± 0.1	14 ± 0.8	3.1 ± 0.1	106 ± 0.8	28.2 ± 0.2	38.0 ± 0.3
CNYBB5R4-286	113.7 ± 6.8	91	20.2 ± 1.0	15 ± 1.0	2.9 ± 0.2	100 ± 12.1	27.6 ± 0.1	35.9 ± 2.6
CNYBB5R4-287	115.3 ± 6.6	91	21.7 ± 0.6	14 ± 0.2	2.8 ± 0.4	103 ± 16.5	26.6 ± 1.6	37.1 ± 8.1
LSD (*p* = 0.05)	9.2	0.6	2.6	3.1	0.7	24.0	1.7	12.1

LSD, least significant difference at 5% probability level.

**Table 3:** Grain quality of parental and five-gene-pyramided BC_3_F_4_ genotypes.

Pyramided Lines	Palatability (*n* = 20)	Protein (*n* = 20)	Amylose (*n* = 20)	Brown Rice (%) (*n* = 20)	Head Rice (%) (*n* = 20)	Total Milled Rice (%) (*n* = 20)
TNG82	76.3 ± 3.2	6.0 ± 0.5	16.0 ± 0.1	77.6 ± 0.2	55.0 ± 0.1	61.5 ± 0.7
IRBB66	60.3 ± 1.1	8.7 ± 0.3	18.1 ± 0.1	72.3 ± 0.1	42.3 ± 0.1	45.7 ± 0.4
CNYBB5R4-272	69.0 ± 2.1	7.4 ± 0.4	14.5 ± 0.9	74.1 ± 0.5	50.5 ± 0.7	57.8 ± 0.8
CNYBB5R4-274	70.0 ± 3.5	7.3 ± 0.7	15.3 ± 0.2	72.8 ± 0.6	31.2 ± 1.8	48.6 ± 0.1
CNYBB5R4-275	69.8 ± 1.1	7.2 ± 0.2	16.0 ± 0.4	75.1 ± 2.3	53.8 ± 1.5	58.7 ± 1.3
CNYBB5R4-276	74.5 ± 2.8	6.4 ± 0.5	15.4 ± 0.1	77.2 ± 0.9	50.1 ± 2.4	59.0 ± 1.0
CNYBB5R4-278	72.3 ± 1.8	6.7 ± 0.3	15.6 ± 0.2	77.5 ± 0.7	55.8 ± 0.3	62.1 ± 0.1
CNYBB5R4-279	72.3 ± 0.4	6.8 ± 0.1	15.3 ± 0.2	77.2 ± 1.8	49.3 ± 2.6	58.1 ± 3.6
CNYBB5R4-285	72.0 ± 1.4	7.0 ± 0.7	15.2 ± 0.1	77.0 ± 1.4	37.0 ± 1.9	54.4 ± 2.2
CNYBB5R4-286	74.3 ± 1.4	6.4 ± 0.3	15.3 ± 0.2	79.3 ± 0.5	48.4 ± 2.4	61.2 ± 0.1
CNYBB5R4-287	74.3 ± 0.4	6.8 ± 0.1	15.4 ± 0.1	75.5 ± 0.5	42.0 ± 9.0	54.7 ± 0.6
LSD (*p* = 0.05)	4.6	0.9	1.0	3.5	9.8	4.5

LSD, least significant difference at 5% probability level.

## Supplementary Materials

The following are available online at https://www.mdpi.com/1422-0067/21/4/1281/s1. Figure S1: The polymorphic markers used for background selection of TNG82/IRBB66 backcross population. Table S1: The genome composition of BC_2_F_2_ derived from TNG82/IRBB66 by 117 polymorphic markers used for MAS.
