# Supplementary material for: Pyramiding Bacterial Blight Resistance Genes in Tainung82 for Broad-Spectrum Resistance Using Marker-Assisted Selection
Source: Int J Mol Sci. 2020 Feb 14;21(4):1281. doi: 10.3390/ijms21041281 (PMC7072918; doi:10.3390/ijms21041281)
Supplement: Supplementary file 1 [file ijms-21-01281-s001.pdf]

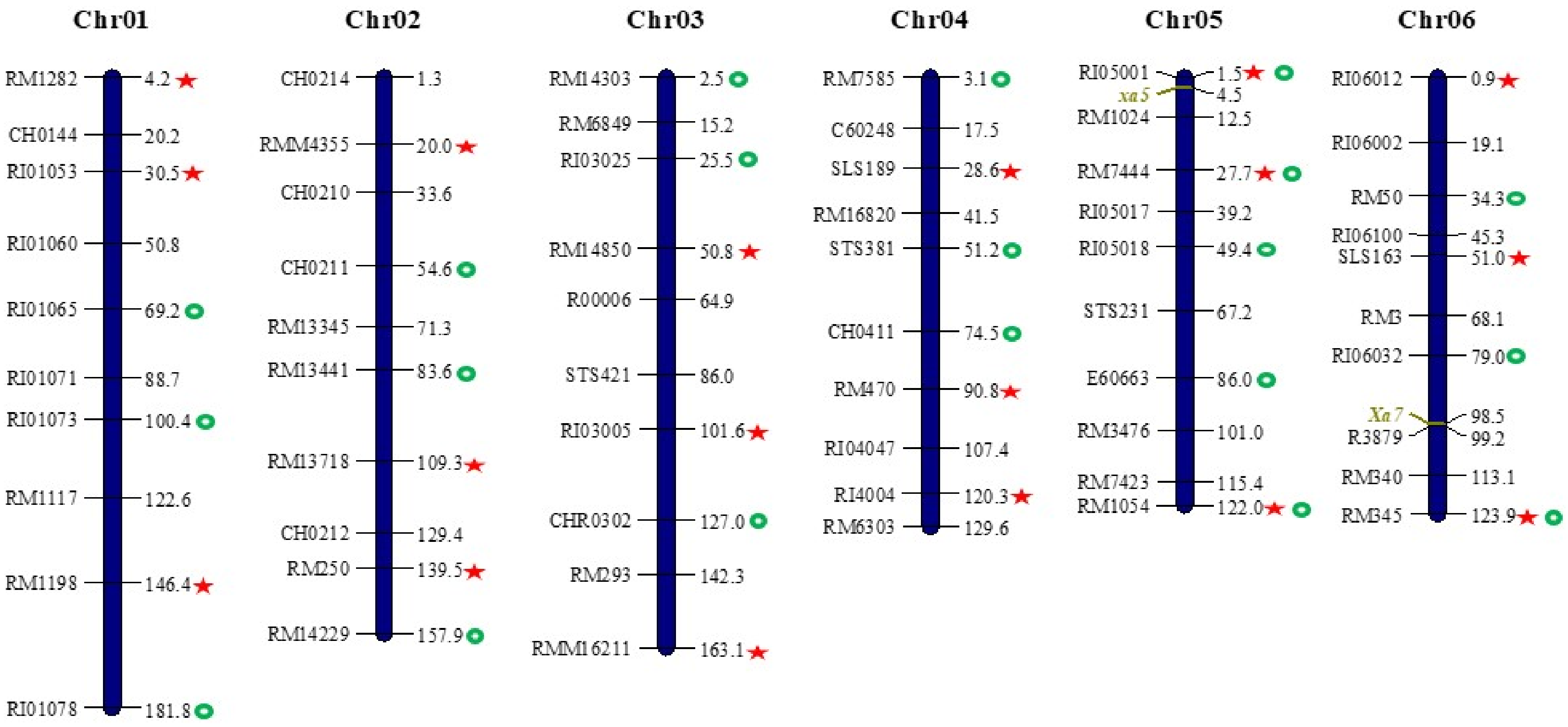
Chr01
RM1282 4.2
CH0144 20.2
RI01053 30.5
RI01060 50.8
RI01065 69.2
RI01071 88.7
RI01073 100.4
RM1117 122.6
RM1198 146.4
RI01078 181.8
Chr02
CH0214 1.3
RMM4355 20.0
CH0210 33.6
CH0211 54.6
RM13345 71.3
RM13441 83.6
RM13718 109.3
CH0212 129.4
RM250 139.5
RM14229 157.9
Chr03
RM14303 2.5
RM6849 15.2
RI03025 25.5
RM14850 50.8
R00006 64.9
STS421 86.0
RI03005 101.6
CHR0302 127.0
RM293 142.3
RMM16211 163.1
Chr04
RM7585 3.1
C60248 17.5
SLS189 28.6
RM16820 41.5
STS381 51.2
CH0411 74.5
RM470 90.8
RI04047 107.4
RI4004 120.3
RM6303 129.6
Chr05
RI05001 1.5
xa5 4.5
RM1024 12.5
RM7444 27.7
RI05017 39.2
RI05018 49.4
STS231 67.2
E60663 86.0
RM3476 101.0
RM7423 115.4
RM1054 122.0
Chr06
RI06012 0.9
RI06002 19.1
RM50 34.3
RI06100 45.3
SLS163 51.0
RM3 68.1
RI06032 79.0
Xa7 98.5
R3879 99.2
RM340 113.1
RM345 123.9

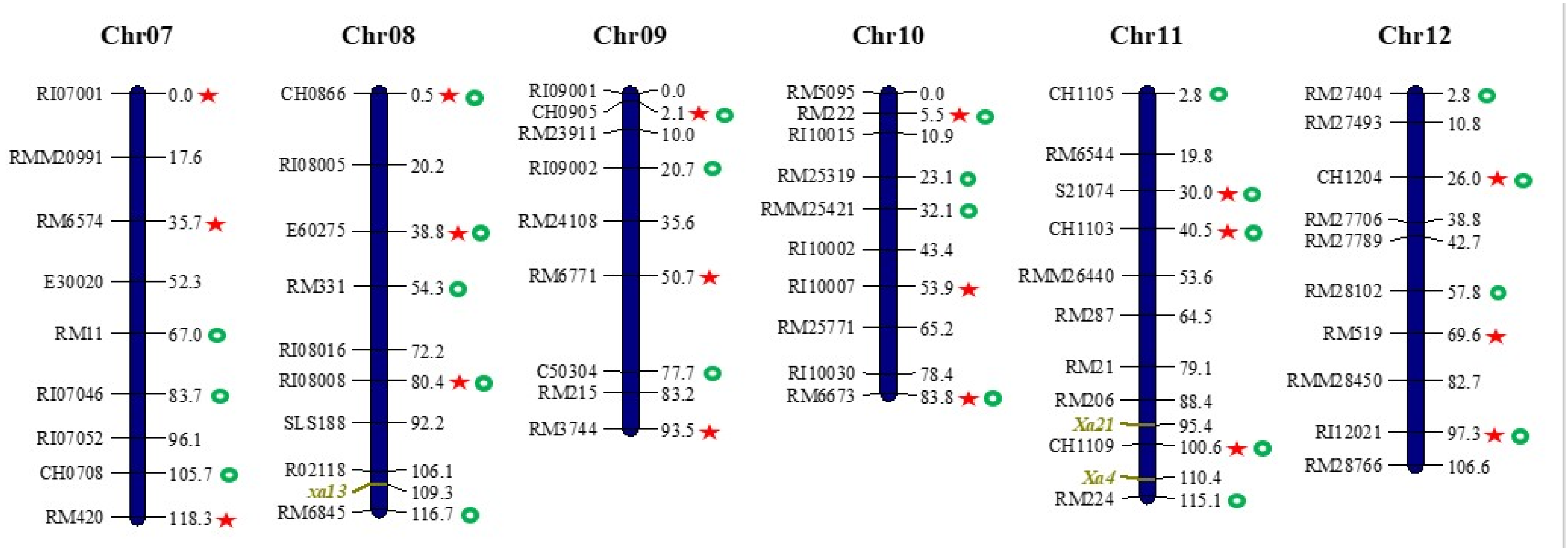

Figure S1. The polymorphic markers used for background selection of TNG82/IRBB66 backcross population. 36 markers among all markers were applied to BC_1F_1 background selection (red star), 44 markers among all markers were applied to BC_2F_1 background selection (green circle), 117 markers among all markers were applied to BC_2F_2 background selection. The prefixes RM indicates SSR markers, C, R, S or E indicates STS markers, and CH, STS, SLS or RI indicates indel markers.

Table S1. The genome composition of BC_2F_2 derived from TNG82/IRBB66 by 117 polymorphic markers used for MAS.

Individual*	A (%)[a]	B (%)[b]	H (%)[c]	N (%)[d]	RPG recovered (%)	Total (cM)	Recombinant[e]	H-segments[f]
TNG82	0	100	0	0	100	1492.8	0	0
44	3.6	93	3.5	0	94.75	1492.8	8	2
21	5.5	92.7	1.8	0	93.6	1492.8	11	2
17	3.1	89.5	7.5	0	93.25	1492.7	9	3
30	4.1	90.1	5.8	0	93	1492.8	11	3
39	5.6	90.9	3.5	0	92.65	1492.8	9	3
40	3.6	88.2	8.2	0	92.3	1492.8	16	7
25	5.6	89.8	4.7	0	92.15	1492.8	8	2
29	6.8	90.9	2.3	0	92.05	1492.8	11	3
14	4.5	88.3	7.2	0	91.9	1492.8	9	4
8	6.6	90.3	3.1	0	91.85	1492.8	10	3
37	6.4	90	3.6	0	91.8	1492.8	13	4
32	4.5	87.9	7.6	0	91.7	1492.8	10	4
24	4.3	87.3	8.4	0	91.5	1492.8	15	4
36	5.2	87.5	7.4	0	91.2	1492.8	12	4
31	5.9	88.1	5.9	0	91.05	1492.7	12	4
10	7.8	87.8	4.5	0	90.05	1492.8	14	2
28	8.5	87.4	4.1	0	89.45	1492.7	13	4
20	8.3	87.1	4.6	0	89.4	1492.8	14	3
23	8	86.1	5.8	0	89	1492.8	11	3
46	8.9	85.2	5.9	0	88.15	1492.8	17	5
3	9.1	84.9	6	0	87.9	1492.8	16	4
22	9.3	85.3	4.5	1	87.55	1492.8	20	5
38	7.3	84	6.7	2.1	87.35	1492.8	18	5
7	6.9	81.4	11.7	0	87.25	1492.8	20	6
35	7.3	83.8	6.3	2.7	86.95	1492.8	23	5
33	9.7	81.4	8.9	0	85.85	1492.8	23	6
1	12.5	83.8	2.9	0.9	85.25	1492.8	20	2
12	9.4	79.4	11.2	0	85	1492.8	23	8
34	13.2	82.7	4.1	0	84.75	1492.8	18	4
45	10.7	79.7	9.7	0	84.55	1492.8	21	7
18	12.2	80.8	7	0	84.3	1492.8	20	5
41	10.5	79	10.5	0	84.25	1492.8	25	8
19	13.1	81.1	5.9	0	84.05	1492.8	18	4
6	7.8	75.6	16.7	0	83.95	1492.8	29	12
5	13.4	80.5	5.2	0.9	83.1	1492.8	23	4
27	11.6	76.4	12	0	82.4	1492.8	28	8
16	10.7	75.2	14.1	0	82.25	1492.8	29	8
15	12	76.4	11.6	0	82.2	1492.8	25	8

Continue Table S1

Individual[*]	A (%)[a]	B (%)[b]	H (%)[c]	N (%)[d]	RPG recovered (%)	Total (cM)	Recombinant[e]	H-segments[f]
13	13.7	77.9	8.4	0	82.1	1492.8	25	8
9	15.3	79	5.7	0	81.85	1492.8	24	4
11	16	79.6	4.4	0	81.8	1492.8	20	3
42	17.2	76.1	6.7	0	79.45	1492.8	28	6
43	15.7	75.3	8.2	0.8	79.4	1492.8	28	7
26	19.2	71.4	9.4	0	76.1	1492.8	26	6
4	19.6	71.5	8.9	0	75.95	1492.8	23	5
IRBB66	100	0	0	0	0	1492.8	0	0

[*] The arrangement was based on the percentage of genetic similarity with TNG82
[a] the percentage of genetic similarity with IRBB66
[b] the percentage of genetic similarity with TNG82
[c] the percentage of heterozygous
[d] the percentage of missing data
[e] the number of chromosome recombination
[f] the number of heterozygous segments in sample
